# Preparation and Properties of New Thermal Reflective Coating for Asphalt Pavement

**DOI:** 10.3390/ma15228087

**Published:** 2022-11-15

**Authors:** Zhenxia Li, Tengteng Guo, Yuanzhao Chen, Chaohui Wang, Qian Chen, Siqing Ding, Qi Chen, Haijun Chen

**Affiliations:** 1 School of Civil Engineering and Communication, North China University of Water Resources and Electric Power, Zhengzhou 450045, China; 2 Henan Province Engineering Technology Research Center of Environment Friendly and High-Performance Pavement Materials, Zhengzhou 450045, China; 3 Zhengzhou City Key Laboratory of Environmentally Friendly High Performance Road and Bridge Materials, Zhengzhou 450045, China; 4 School of Highway, Chang’an University, Xi’an 710064, China

**Keywords:** asphalt pavement, thermal reflection coating, filler modification, cooling performance

## Abstract

**Highlights:**

**What are the main findings?**

- A new heat reflective coating material for asphalt pavement was obtained.
- The inorganic filler with lipophilicity after surface modification was obtained.
- A double coating structure with better cooling effect is obtained.
- A heat reflective coating with excellent road performance was obtained.

**What is the implication of the main finding?**

- Compared with the traditional heat reflection coating, it has better physical and chemical properties and lower cost.
- Make the inorganic filler better dispersed in the matrix.
- The higher the temperature, the better the cooling effect of the coating.
- After the heat-reflective coating is laid, its skid resistance, wear resistance and impermeability meet the requirements of the specification, and the performance is improved.

**Abstract:**

This paper aims to study the applicability of an epoxy resin modification to improve its anti-aging properties, which are conducive to road performance. To achieve this goal, a wide range of laboratory activities were conducted, including an emulsion mixed with epoxy resin and liquid phenolic resin as the coating substrate; surface-modified titanium dioxide, silica, hollow glass beads and sericite powder as functional fillers; then adding pigments and various additives to prepare a new asphalt pavement heat-reflective coating. Secondly, the optimum brushing amount of the coating was obtained, and the cooling effect was clarified. Finally, the road performance was evaluated by testing the coating’s skid resistance, wear resistance and impermeability. The results show that the skid resistance, abrasion resistance and impermeability of the heat reflection coating meet the specification requirements.

## 1. Introduction

Given the excellent smoothness and driving comfort of asphalt pavement and its short construction time, less driving noise and dust, and simple and fast repair and maintenance, it occupies nearly 90% of the total mileage of high-grade highways [1]. However, asphalt pavement also has the characteristics of easy heat absorption. High temperatures will cause the adhesion force between the binder of asphalt pavement and mineral aggregate to decrease, the fluidity to increase, the creep property to increase, and the dynamic stability to decrease [2]. Under heavy and repeated loads, the shear stress and shear flow in the layer will be generated, resulting in the deformation of the pavement. In addition, the accumulation of deformation caused by road traffic channelization will produce damage, such as rutting and congestion [2,3]. The thermal reflective coating can reduce the road surface temperature by increasing the road surface reflectivity, namely reducing the absorbed radiation energy [4]. It can radiate the energy radiated to its surface through the window with a wavelength range of 2.5–15 μm to the outside world to actively limit the temperature rise of the road surface and reduce the internal and surrounding temperature of the coating under the condition of no energy consumption.

Anna et al. [5] prepared a common color road solar thermal reflective coating. Through the analysis of the reflective spectrum of the coating by factors such as filler particle size and binder, it was found that the reflectivity of the coating on the whole band could reach about 20%. Xie et al. [6] prepared a thermal reflection coating using several common fillers, and the infrared reflectivity was close to 50%, and the total reflectivity reached 34.85%. TiO_2_ had the highest contribution to the cooling capacity of all fillers. Still, with the increase in TiO_2_ content, the reflectivity of the coating only increased in the infrared region and even decreased slightly in other bands. Kyriakodis et al. [7] coated the thermal reflective coating on a large area of asphalt pavement that covered an area of 0.037 km^2^. Through a series of tests, the road surface temperature was reduced by 11.5 °C, and the ambient temperature was reduced by 1.5 °C. At the same time, the ease of vision and driving greatly improved. 

However, the aging phenomenon of the coating during service may weaken about half of its cooling capacity. Carnielo et al. [8] and Rossi et al. [9] found that applying a thermal reflection coating can effectively reduce the temperature of asphalt pavement, which has great potential to alleviate the ‘urban heat island effect’. Uemoto [10] points out that the reflectivity of the coating is inversely proportional to its color depth; that is, the lower the depth of color, the better the cooling performance of the thermal reflection coating, but too shallow will lead the road surface to produce dazzling, and other hazards to traffic safety have brought great hidden dangers, so blind to the color of the coating is not a good choice to reduce road temperature. Synnefa et al. [11] studied five different color coatings used on asphalt pavement by adding different coloring pigments and found that as long as the reflectivity of the coating was between 0.27 and 0.55, it was higher than that of the asphalt pavement in any color. All five coatings could achieve the purpose of reducing pavement temperature, among which the best one could reduce pavement temperature by 12 °C. Tukiran et al. [12] studied the thermal performance of five kinds of thermal reflective coatings on the market with different colors by measuring and evaluating the cooling capacity and reflectivity. The results showed that all color coatings could improve the cooling performance of asphalt pavement to varying degrees, and the reflectivity and cooling value of the white coatings reached 61% and 17 °C, respectively. The lowest green coatings were 14% and 1.26 °C, respectively. Du et al. [13,14,15] studied the pavement surface temperature after painting the coating on different asphalt pavement structures. The results showed that different surface composite structure designs could reduce the pavement temperature by 2–8 °C, and there was also a cooling of about 0.5–5 °C inside the road. Zheng et al. [16] studied the relationship between road surface temperature and human thermal comfort in urban areas by establishing the near-surface thermodynamic model of the road surface. The results showed that after the thermal reflection coating was laid, the radiation heat of the road surface was reduced by nearly 70%, and the convective heat was reduced by nearly 50%.

Moreover, the road surface and near-surface temperatures were decreased, which greatly improved human comfort. Chen Qian et al. [17] tested the cooling capacity of various coatings and simulated the complex coupling environment affected by temperature, traffic load and pollution in the actual environment. The durability evaluation index of road cooling coatings under complex working conditions was proposed and evaluated by a multi-objective decision-making method. Shen Aiqin et al. [18] used a self-made cooling simulation device to explore the influence of coating amount and temperature on the indoor cooling effect of the coating after preparing a thermal reflection coating and carried out an outdoor cooling test. In terms of the consistency of other parameters, the surface temperature of different specimens in the indoor test had a stronger correlation, which had a better reference value.

Most of the existing asphalt pavement thermal reflection coatings are based on epoxy resin, and epoxy resin has many advantages, but it is brittle and ages easily. In this paper, a suitable amount of liquid phenolic resin was added to epoxy resin, and the active groups on the resin chain were combined by copolymerization to toughen and improve the aging resistance. Then, a new type of asphalt pavement thermal reflective coating was prepared with the mixed resin as the thermal reflective coating substrate, titanium dioxide, hollow glass beads, silicon dioxide and sericite powder as functional fillers, ferric oxide red as a pigment, propylene glycol and other materials as additives, and quartz sand for its anti-skid particles. In this paper, in the process of preparation and technical research of heat reflective asphalt pavement coating, using the principle of coating cooling, according to the characteristics of sunlight and the principle of cooling, the influencing factors of the cooling effect were obtained, and then the mix ratio design and coating preparation were carried out. Then, through the indoor and outdoor cooling tests for data analysis, the road performance was tested, and the environmental performance was evaluated. Finally, exploration of the construction process resulted in a new type of asphalt pavement heat-reflective coating.

## 2. Coating Material

### 2.1. Composition of Raw Materials

Asphalt pavement thermal reflection coating comprises a matrix, functional fillers, pigments, additives and anti-skid particles.

(1) Elementary body

The selection of matrix resins should avoid subs containing O = C-, -OH, C-O-C- as much as possible [19]. After repeated comparison and selection, the non-solvent E51 (618) epoxy resin was used as the resin of this experiment, and the 593 epoxy curing agent was used as the curing and plasticizer material of this coating. The liquid phenolic resin was added to the coating to toughen and modify the epoxy resin, and the mixed resin was used as the coating matrix material. After many tests, it was found that when the mass ratio of epoxy resin to liquid phenolic resin in 100 g mixed resin was 97: 3. The basic characteristics of each material are shown in Table 1, Table 2 and Table 3.

(2) Functional fillers

**Figure 1:**
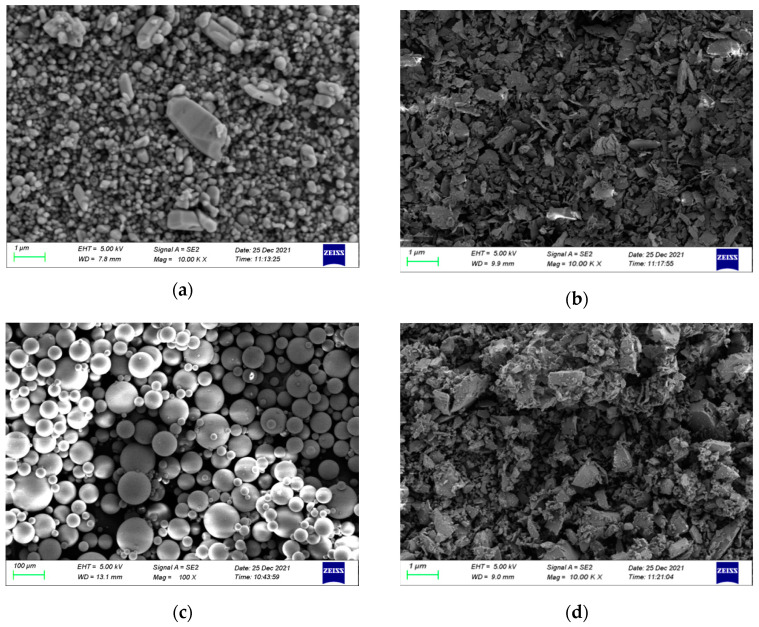
Micromorphology of functional fillers. (**a**) Titanium dioxide. (**b**) Silicon dioxide. (**c**) Hollow glass microspheres. (**d**) Sericite powder.

The functional fillers selected in this paper are rutile titanium dioxide, hollow glass beads, silica and sericite powder. The main characteristics of each material are shown in Table 4, and the microstructure is shown in Figure 1.

(3) Pigment

The refractive index of epoxy resin is 1.48, while iron oxide red has a high refractive index of 2.8. At the same time, the light red color shown after its incorporation into the coating can bring the driver vigilance and concentration after laying. Based on safety considerations, iron oxide red was selected as pigment to be added to the coating solution.

(4) Additive

To improve the properties of the coating solution and weaken its negative influence on some road performance of asphalt pavement, fumed silica, propylene glycol, and active diluent, which can participate in the reaction during coating preparation, are added as additives in addition to the matrix and functional filler.

(5) Anti-slip pellet

Soft anti-skid particles have low hardness and are often prone to deformation due to vehicle load when used in the lane, which makes them unable to provide greater friction; hard anti-skid particles usually use inorganic material particles. When making contact with rubber tires, such anti-skid particles cause deformation of the rubber tires due to their high hardness, so the contact surface between tires and the ground can be widened to better achieve anti-skid properties. This paper used hard anti-skid particle quartz sand.

### 2.2. Surface Modification of Functional Filler

**Figure 2:**
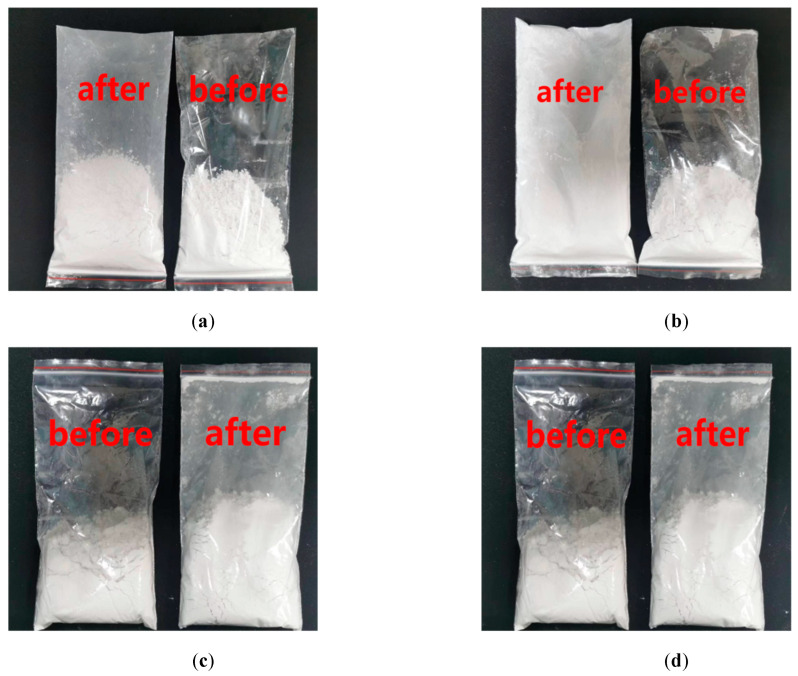
Appearance of functional fillers before and after surface modification. (**a**) Titanium dioxide, (**b**) Silicon dioxide, (**c**) Hollow glass microspheres, (**d**) Sericite powder.

To ensure the functional filler particles have more affinity with the base material, silane coupling agent KH-550 was used to modify the surface of these functional fillers. This agent can help reduce the surface energy of particles so that they can be stable and evenly dispersed in the asphalt pavement thermal reflection coating and give full play to the cooling effect of the coating. The appearance of titanium dioxide before and after modification is shown in Figure 2.

Figure 2 shows that the plastic packaging bag adsorbs a lot of modified inorganic fillers, but the unmodified fillers are not adsorbed. This is because the surface of the filler is mainly composed of inorganic groups without surface modification, which is manifested as hydrophilic and hydrophobic. The plastic bag is composed of organic compounds, so there is no good combination between the two. When modifying the surface of inorganic fillers, some polar groups in the modifier are adsorbed with them. The other part is oriented towards the organic groups in the resin solution, which is equivalent to wearing a layer of ‘camouflage clothing‘ on the surface of the fillers. This ensures a good combination of the modified filler and the plastic packaging bag.

### 2.3. Determination of Filler Content

With the increase in the proportions of a functional filler and matrix resin, the cooling effect of the coating was effective, but the rising trend tends to be gentle after exceeding a certain limit. The increase in the surface area means the resin cannot completely fill the gaps between the pigments and fillers, and there will be many defects after the coating, decreasing the reflection ability and the instability of the particles. When the proportion is small, the appearance of smearing on the asphalt pavement is not uniform, and the covering force is insufficient and therefore does not yield a good cooling effect. According to multiple tests, the cooling effect and operability are good when the filler content accounts for 25–35% of the mass of the mixed matrix.

(1) TiO_2_ dosage

**Figure 3:**
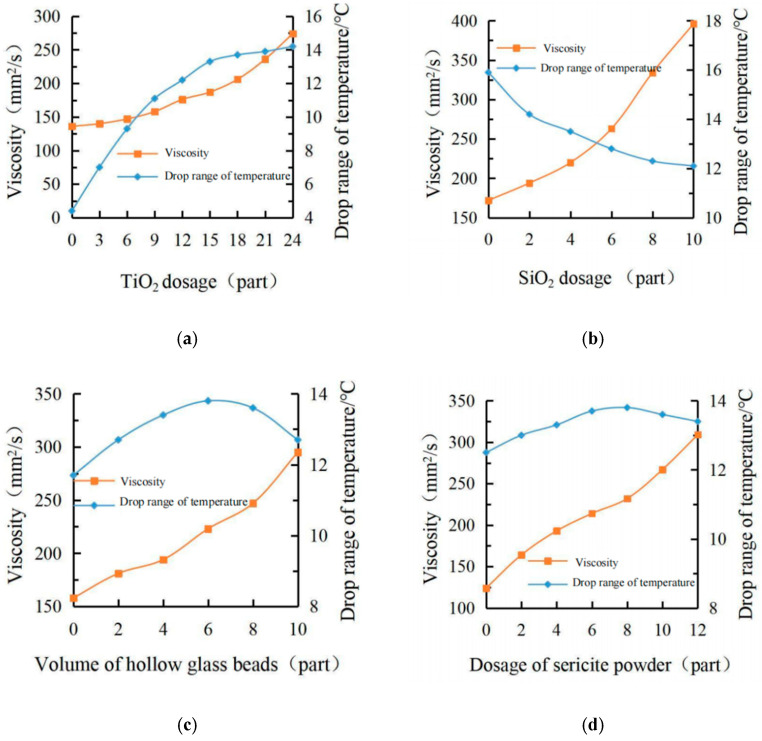
Effect of different fillers content on cooling value and viscosity. (**a**) Effect of different TiO2 contents on coatings, (**b**) Effect of different SiO2 content on coatings, (**c**) Effect of different content of hollow glass beads on Coatings, (**d**) Effect of different dosages of sericite powder on coatings.

Under the fixed conditions of 100 parts of the matrix resin, 4 parts of SiO_2_, 6 parts of hollow glass beads and 6 parts of sericite powder, the influence of the TiO_2_ content on the partial properties of the coating was analyzed, and the results are shown in Figure 3a. It can be seen that the viscosity increased slowly, and the cooling capacity increased rapidly when the TiO_2_ content was low. With the increase in TiO_2_ content, the viscosity gradually increased, and the cooling value decreased. TiO_2_ had a significant influence on the cooling effect of the coating, and the cooling value reached 13.7 °C when the amount of TiO_2_ was 18 phr. When the dosage continued to increase, the increase in the cooling value tended to be flat, and the viscosity value of the coating increased significantly, which is not conducive to construction. The dosage of TiO_2_ is 15 ~ 18 phr.

(2) SiO_2_ dosage

Under the fixed conditions of a 100 phr matrix resin, 18 phr TiO_2_,6 phr hollow glass beads and 6 phr sericite powder, the effect of the SiO_2_ content on the performance of the coating was judged, as shown in Figure 3b. When the SiO_2_ was added, the cooling value of the coating decreased greatly. With the increase in SiO_2_ content, the decreasing trend of the cooling value of the coating gradually becomes uniform, which is because SiO_2_ can make the coating surface rougher and increase the heat absorbed by the coating. The viscosity of the coating increased, and the increase rate was relatively slow in the early stage. When the dosage was more than 4 phr, the viscosity increased rapidly. So, the dosage of SiO_2_ was 2 ~ 4 phr.

(3) The volume of hollow glass beads

Under the fixed conditions of 100 portions of the matrix resin, 18 portions of TiO_2_, 4 portions of SiO_2_ and 6 portions of sericite powder, the effect of the hollow glass microsphere content on the coating performance was analyzed, and the results are shown in Figure 3c. It can be seen from the figure that the hollow glass microsphere had a significant influence on the viscosity of the coating: when the dosage was less than 4 phr, the viscosity generally showed a slow increase trend, but with the continuous increase of the dosage, the viscosity of the coating increased sharply. The effect of the beads on temperature did not just increase or decrease the cooling capacity but also strengthened the cooling effect at a relatively small dosage and weakened the cooling effect after more than 6 phr. This may be due to the addition of too many hollow glass beads will change its arrangement, and too large a volume to cover other fillers, resulting in a decline in its cooling capacity considering the content of 4 ~ 6 phr.

(4) Dosage of sericite powder

Under the fixed conditions of 100 portions of the matrix resin, 18 portions of TiO_2_, 4 portions of SiO_2_, and 6 portions of hollow glass beads, the influence of the amount of sericite powder on the coating performance was judged, and the results are shown in Figure 3d. The results show that sericite powder had little effect on temperature, but it had a slight increase in 0 ~ 8 phr and a large increase in 4 ~ 8 phr, and a slight decrease after more than 8 phr. This is because the ‘maze effect‘ can be formed due to the sericite powder’s large diameter–thickness ratio, which weakened the heat transfer process to a certain extent. However, when the dosage was too large, it will gradually accumulate and cover up the other functional fillers, resulting in other fillers that cannot display the cooling effect. With the increase in the sericite powder content, the viscosity of the coating first showed a fast trend, then slow, and then fast again. However, the second rapid increase in the viscosity of the coating after the content of sericite powder exceeded 8 phr led to the difficulty of a subsequent coating. Therefore, the content of sericite powder was 4 ~ 8 parts.

Through the calculation of the standard deviation, it can be seen that the standard deviation of the cooling value of different TiO_2_, SiO_2_, hollow glass beads and sericite powder is 3.25, 1.30, 0.71 and 0.42, respectively. It can be concluded that the most influential factor on the cooling value is the amount of TiO_2_, and the standard deviation of viscosity is 44.28, 79.18, 45.32 and 57.56, respectively. It can be seen that the most influential factor in viscosity is the amount of SiO_2_.

The orthogonal test was carried out with the coating’s viscosity, gloss and cooling value as the evaluation indexes. A total of nine groups of experiments were designed by four factors and three levels of the orthogonal table, as shown in Table 5. The test results are shown in Table 6. In the test, the matrix resin was fixed at 100 parts, and the amount of each filler was the corresponding proportion of the matrix amount.

**Figure 4:**
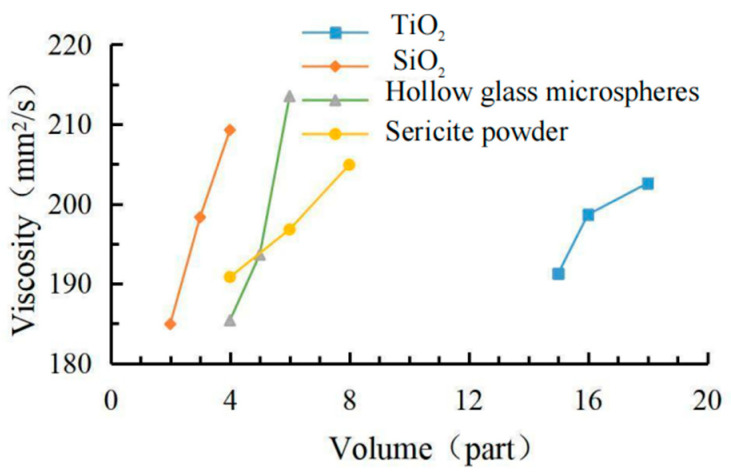
Factor effect of viscosity.

**Figure 5:**
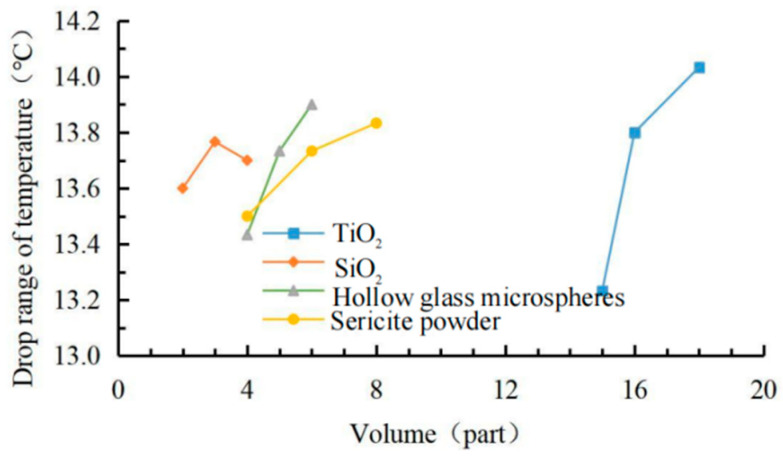
Factor effect of cooling value.

**Figure 6:**
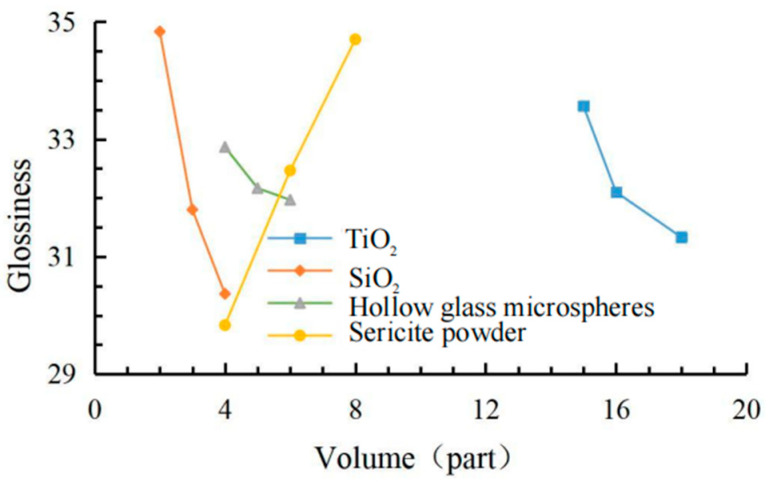
Factor effect of glossiness.

The data in Table 6 shows the effects of the coating viscosity, cooling value and gloss factor shown in Figure 4, Figure 5 and Figure 6. The comprehensive analysis method was used to select the most balanced ratio of each performance. According to the orthogonal test results, the coating with 18 phr TiO_2_ had the best cooling effect. Compared with 16.5 phr TiO_2_, the maximum cooling value increment was not large, but the viscosity growth rate was low, and the gloss of the coating also decreased slightly. When the SiO_2_ content was 2 ~ 4 phr, the gloss of the coating decreased continuously, but when the SiO_2_ content was 3 ~ 4 phr, the viscosity increased greatly, and the cooling value decreased. Hollow glass beads have a small improvement on the gloss of the coating, and the increase of its content had a significant impact on the cooling value, but when the content is greater than 5 phr, the viscosity began to increase significantly, and the increase of the cooling value decreased. The sericite powder was mainly aimed at improving the physical and chemical properties of the coating. The addition of sericite powder slightly increased the maximum cooling value of the coating but also increased the viscosity and gloss of the coating, but the growth rate was not large in the range of 4 ~ 6 phr. In conclusion, under the condition of 100 phr matrix resin, 18 phr modified TiO_2_, 3 phr modified SiO_2_, 5 phr modified hollow glass beads and 6 phr modified sericite powder can make the coating achieve the best balance among the three indexes.

### 2.4. Determination of Pigment Content

**Figure 7:**
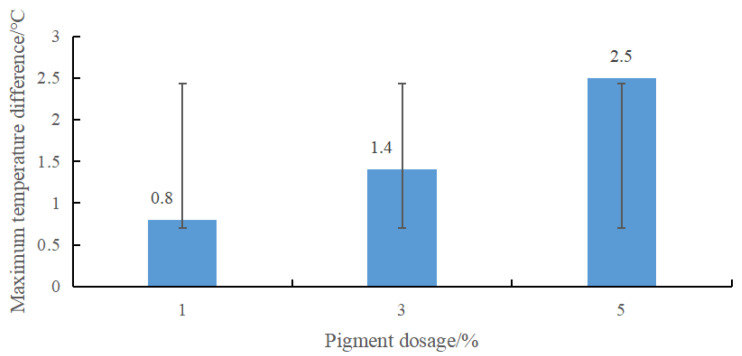
Temperature difference value of different pigment content.

The cooling test determined the pigment content. The three groups of pigment content of 1%, 3% and 5% of the matrix mass were set as the test group, and the white coating without pigment was the control group. The cooling test measured the temperature difference. The results are shown in Figure 7.

It can be seen from Figure 7 that the temperature difference between the experimental group and the control group increased with the increase in pigment content. When the content was from 1% to 3%, the temperature difference was 0.6 °C. When the content was from 3% to 5%, the relative temperature difference was large, reaching about 1.1 °C, which significantly weakened the cooling capacity of the thermal reflective asphalt pavement coating. Considering the pavement color was shallow when the dosage was 1%, which is not conducive to driving, the dosage of ferric oxide red was selected as 3%, and the temperature difference with the white coating was 1.4 °C.

### 2.5. Material Dosage of Each Component

The determined amounts of thermally reflective asphalt pavement coating materials are shown in Table 7:

## 3. Test Scheme

### 3.1. Indoor Cooling Test

(1) Determination of coating amount

After evenly dividing a rutting plate specimen into four parts, an electric drill was used to drill in parallel at a distance of 2 cm from the upper surface of each part of the specimen. The aperture was about 0.3 ~ 0.5 cm, and the hole depth was about 7 ~ 8 cm. The thermocouple temperature sensor was arranged inside. The four parts were coated with 0 kg/m^2^, 0.5 kg/m^2^, 0.8 kg/m^2^ and 1.1 kg/m^2^ heat-reflective asphalt pavement coatings, respectively. The bottom of the rutting plate was paved with foam board and wrapped with tin foil to avoid the test error caused by heat loss. After the coating on the rut board specimen solidified, an iodine tungsten lamp was used to simulate the sunlight for the cooling test. The surface temperature and internal temperature of the rutting plate before the test was measured and recorded as 25.4 °C and 24.7 °C, respectively, which were used as the starting temperature of the rutting plate test. The temperature of the four parts was measured every 5 min for a total of 12 times.

(2) Determination of coating structure

**Figure 8:**
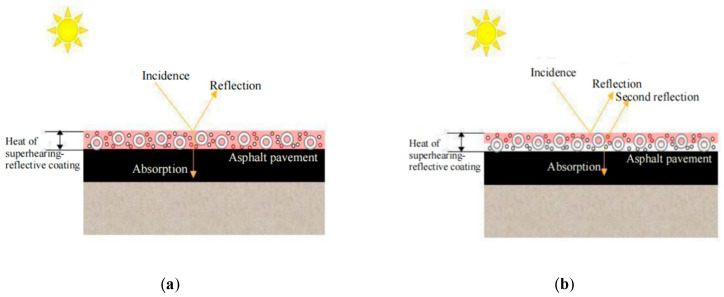
Working principle of coatings with different structures. (**a**) Single-layer coating structure, (**b**) Double-layer coating structure.

The working principle of the double-layer coating structure is shown in Figure 8. It can be seen from Figure 8 that the double-layer brush structure was used to reflect the solar radiation transmitted through the surface layer again at the bottom layer, thereby increasing the number of reflections of the incident light in the coating. At the same time, the white coating was used as the bottom layer to force the pavement and further improve the cooling effect of the coating.

The concrete implementation steps are as follows: The white coating without pigments was used as the bottom coating on the asphalt pavement, and the coating with iron oxide red as the pigment was used as the surface layer after the coating was uniformly cured. Three kinds of two-layer structure coating dosage schemes are: (1) Test group 1: bottom coating amount is 0.2 kg/m^2^, surface coating amount is 0.6 kg/m^2^; (2) Test group 2: bottom coating amount was 0.4 kg/m^2^, surface coating amount was 0.4 kg/m^2^; (3) Test group 3: bottom coating amount was 0.6 kg/m^2^, surface coating amount was 0.2 kg/m^2^; the cooling capacities of the three groups of coating on the rutting plate specimens were compared with that of the single-layer coating structure, and the temperatures of the four groups were measured 12 times every 5 min.

### 3.2. Outdoor Cooling Test

The outdoor cooling test was carried out to evaluate the coating’s cooling capacity more comprehensively. Firstly, the coating was brushed on the asphalt pavement with a coating amount of 0.8 kg/m^2,^ and its geometric center was determined as the temperature to be measured. A point on the same road section was selected as the control point. At 10:00 on the test day, the ambient temperature was 32 °C, and the surface temperature of the uncoated specimen was 37.2 °C. The surface temperature of the coated part was 35.1 °C, which was taken as the initial temperature of the test. The surface temperature was measured every 0.5 h between 10:00 and 16:00 for a total of 12 measurements.

### 3.3. Anti-Skid Test

Adding 0.2 kg/m^2^ quartz sand as anti-skid particles, the coating adopted the ‘sandwich’ structure that is the coating bottom + anti-skid particles + coating surface coating structure. According to the ‘Highway Subgrade Pavement Field Test Regulations’ (JTG 3450-2019), three measuring points without coating were selected as the control group, and three measuring points with coating were selected as the experimental group. The pendulum friction instrument was used for the control test. Each measuring point was repeated five times, and the average value was taken as the road anti-skid value of the point.

### 3.4. Structural Depth Test

On the asphalt pavement, three measuring points without coating were selected as the control group. Three measuring points with coating added with anti-skid particles were selected as the experimental group. An electric sanding instrument measured the structural depth.

### 3.5. Wear Resistance Test

According to the ‘Highway Engineering Asphalt and Asphalt Mixture Test Procedures’ (JTG E20-2011), six corresponding specimens were made. The specimens were baked in the oven at a temperature of 60 °C for 16 h. After drying, three specimens were randomly selected to be painted with a 0.8 kg/m^2^ coating solution on their surface, and then the wet wheel wear comparison test was carried out with the specimens without a coating solution. The mass of each specimen before and after the test was weighed, the mass change was compared, and the wear mass loss was calculated according to Formula (1). Three groups of parallel tests were carried out.

(1) $$W=\left(M_{a}-M_{b}\right)/A$$

Formula: *M*_a_−quality of the specimen before wear; *M*_b_−The quality of the specimen after wear; *A*−Wear area of rubber tube of wear head, namely 0.03077514 m^2^; *W*−Loss of wear quality.

### 3.6. Water Impermeability Test

Referring to the ‘Highway Subgrade and Pavement Field Test Procedure’ (JTG 3450-2019), the seepage coefficients of five rut board specimens before and after coating were measured.

## 4. Results Analysis and Discussion

### 4.1. Evaluation of Indoor Cooling Effect

(1) Determination of coating amount

**Figure 9:**
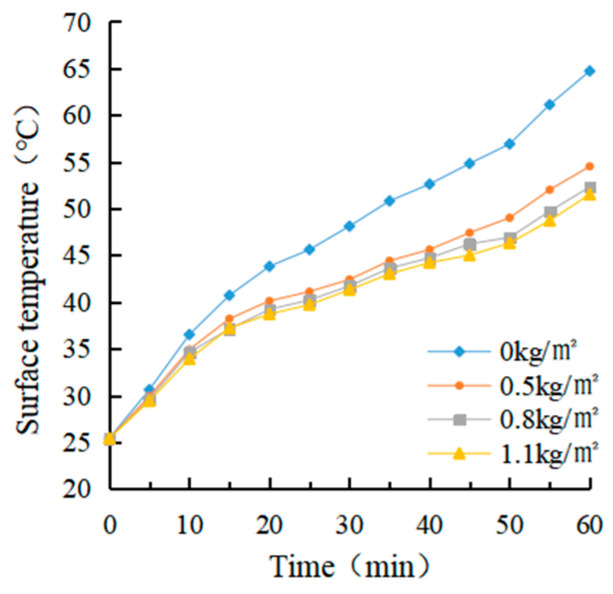
Surface temperature diagram.

**Figure 10:**
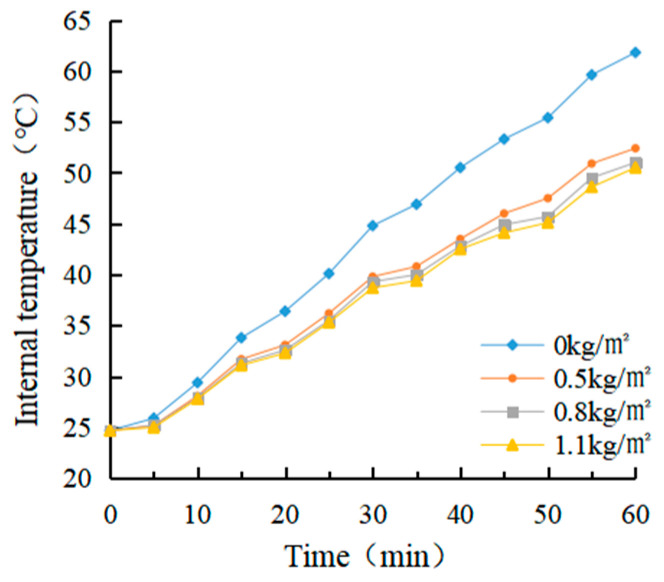
Internal temperature diagram.

**Figure 11:**
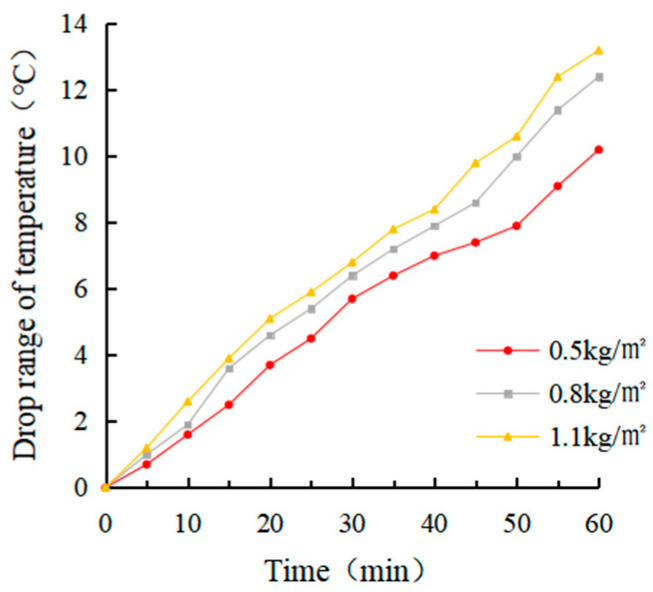
Surface cooling value diagram.

**Figure 12:**
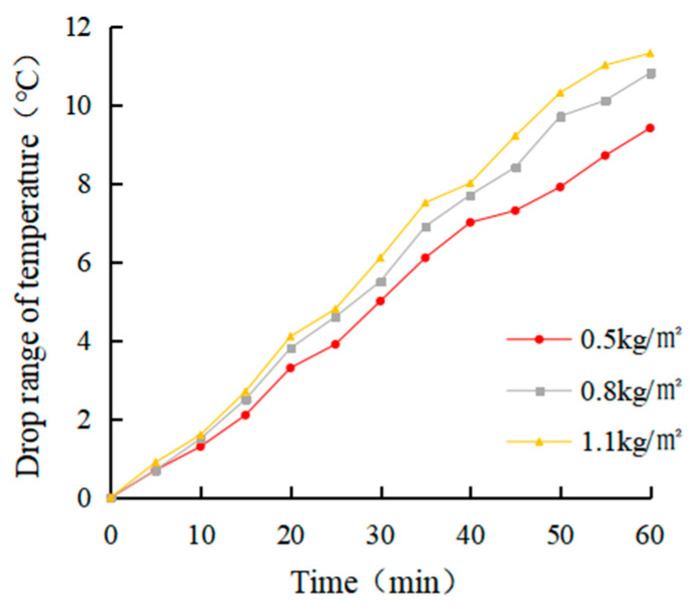
Internal cooling value diagram.

The real-time temperature diagram and cooling value diagram of the asphalt surface and internal are shown in Figure 9, Figure 10, Figure 11 and Figure 12. It can be seen that the internal and external cooling values of the thermal reflection coating are proportional to the internal and external temperature of the specimen and the coating amount. The maximum surface cooling values were 10.2 °C, 12.4 °C and 13.2 °C, respectively, when the coating amount was 0.5 kg/m^2^, 0.8 kg/m^2^ and 1.1 kg/m^2^; the maximum internal cooling values were 9.4 °C, 10.8 °C and 11.3 °C, respectively. When the amount of coating increased from 0.5 kg/m^2^ to 0.8 kg/m^2^, the maximum surface temperature of the specimen without coating was 64.7 °C, and the internal temperature was 61.8 °C, the maximum surface cooling value increased by 21.6%. The maximum internal cooling value increased by 14.9%. When 0.8 kg/m^2^ increased to 1.1 kg/m^2^, the cooling capacity was not obvious, and the maximum cooling value of surface and internal increased by 6.5% and 7.4%, respectively. At 60 min, the internal temperature of the specimen without coating was 2.9 °C lower than that of the surface temperature. In comparison, the internal temperature of the specimen with coating was 2.1 °C, 1.3 °C and 1.0 °C lower than that of the surface temperature, respectively. This is because the specimen without a coating caused a certain loss in heat transfer. With the increase of the coating amount, the heat loss of the specimen with coating was increased, and its reflection and heat insulation ability were also enhanced. However, when the coating amount increased from 0.5 kg/m^2^ to 0.8 kg/m^2^, the internal and external temperature difference was significant at 38.1%. When 0.8 kg/m^2^ increased to 1.1 kg/m^2^, the decrease was 23.1%. Therefore, it is recommended that the coating amount be 0.8 kg/m^2^, and the internal and external temperature difference with the uncoated specimen was 12.4 °C and 10.8 °C, respectively. 

It can be seen from the calculation of standard deviation that when the brushing amount is 0 kg/m^2^, the standard deviation of the surface and internal temperature of the specimen with the change of illumination time is the largest, which is 11.15 and 12.11, respectively. The standard deviation of each group decreases with the increase of brushing amount. This shows that the laying of the coating can effectively block heat transfer. When the coating amount was 1.1 kg/m^2^, it had the greatest influence on the specimen’s surface and internal cooling value. As the coating amount increased, the standard deviation showed an increasing trend, which indicates that the coating can effectively cool the surface and interior of the asphalt.

(2) Determination of coating structure

The real-time temperature and cooling values of the surface and interior of each group are shown in Figure 9, Figure 10, Figure 11 and Figure 12.

It can be seen from the calculation of standard deviation that when the brushing amount is 0 kg/m^2^, the standard deviation of the surface and internal temperature of the specimen with the change of illumination time is the largest, which is 11.15 and 12.11, respectively. The standard deviation of each group decreased with the increase of the brushing amount. This shows that the laying of the coating can effectively block heat transfer. When the coating amount was 1.1 kg/m^2^, the standard deviation of the specimen’s surface and internal cooling value with time was the largest, which were 3.99 and 3.78, respectively. With the increase in the coating amount, the standard deviation showed an increasing trend, which indicated that the coating could effectively cool the surface and interior of the asphalt.

**Figure 13:**
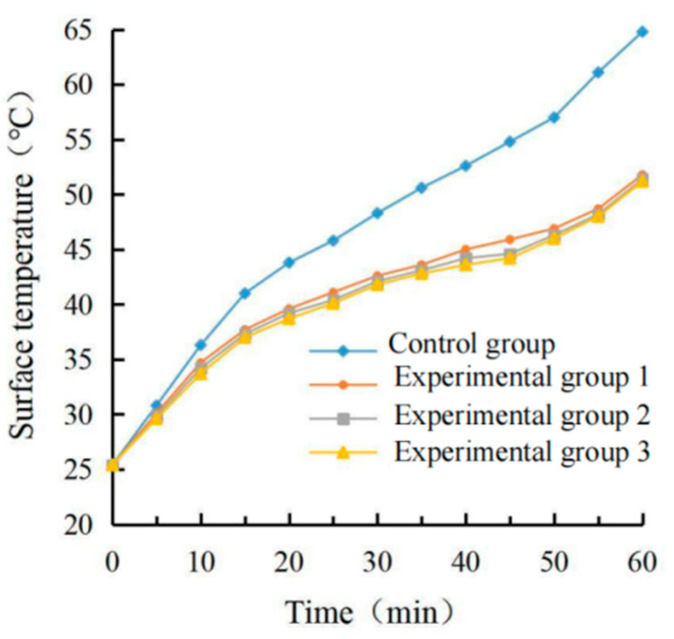
Surface temperature diagram.

**Figure 14:**
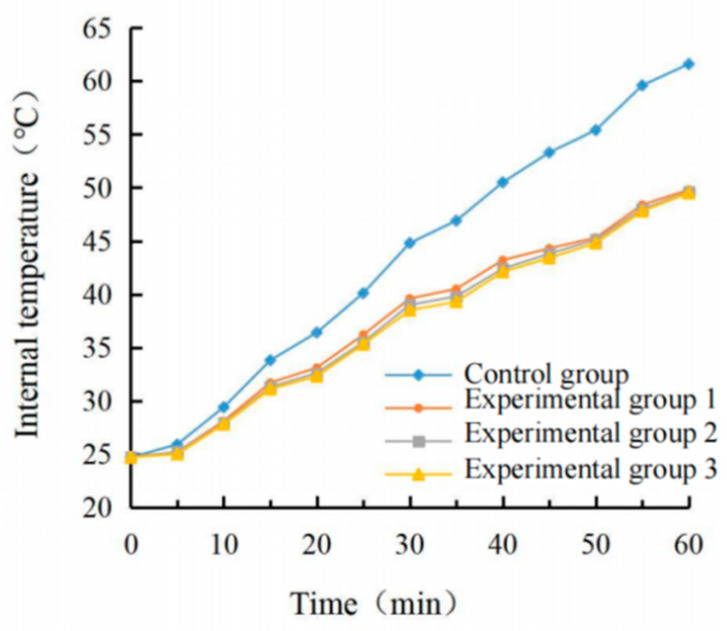
Internal temperature diagram.

**Figure 15:**
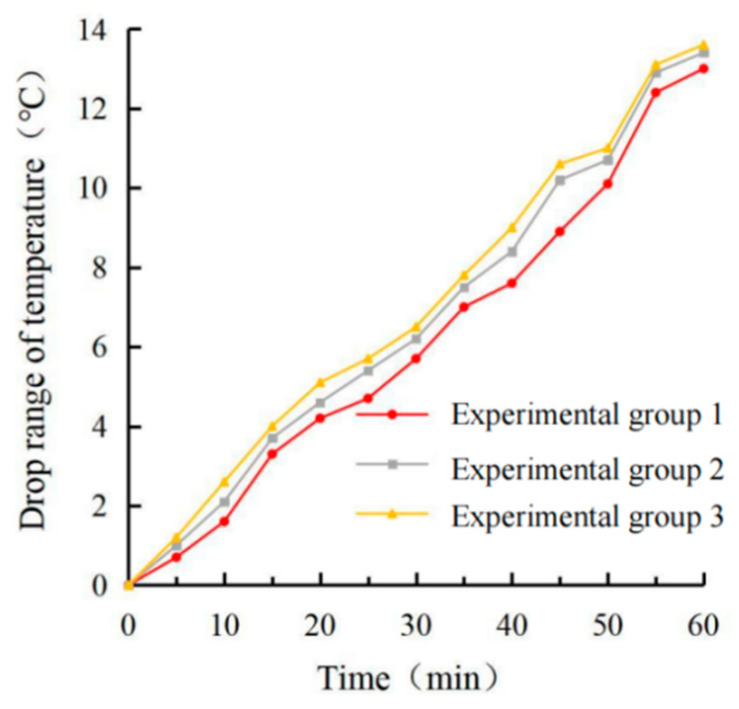
Surface cooling value diagram.

**Figure 16:**
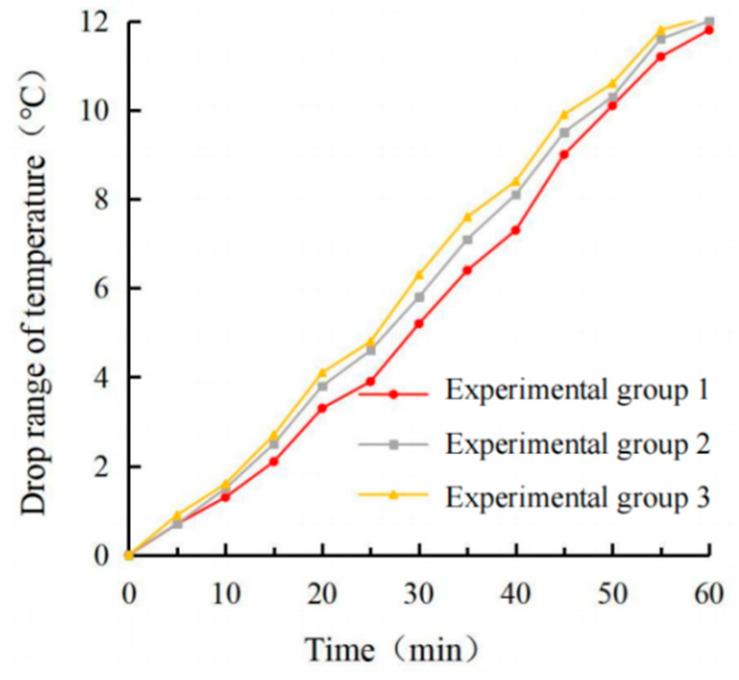
Internal cooling value diagram.

It can be seen from Figure 13, Figure 14, Figure 15 and Figure 16 that the maximum surface and internal cooling values of the three schemes are 13.0 °C, 11.8 °C; 13.4 °C, 12.0 °C; 13.6 °C, 12.1 °C, respectively. Compared with the single-layer coating structure of 12.4 °C, 10.8 °C, the cooling value growth rates were 4.8%, 9.3%; 8.1%, 11.1%; 9.7%, 12.1%. Although the cooling capacity of experimental group 3 reached the highest, the growth rate was extremely limited, and the pavement color was lighter, which could easily cause adverse effects such as glare. Through the calculation of the standard deviation, it can be seen that the coating’s surface temperature and internal temperature changed the most with time in test group 1, and the coating’s surface cooling and internal cooling changed the most with time in test group 3. Therefore, test group 2 had the least response to light, so the brushing method adopts the double-layer coating structure with the bottom brushing amount of 0.4 kg/m^2^ and the surface brushing amount of 0.4 kg/m^2^ in test group 2.

### 4.2. Evaluation of Outdoor Cooling Effect

**Figure 17:**
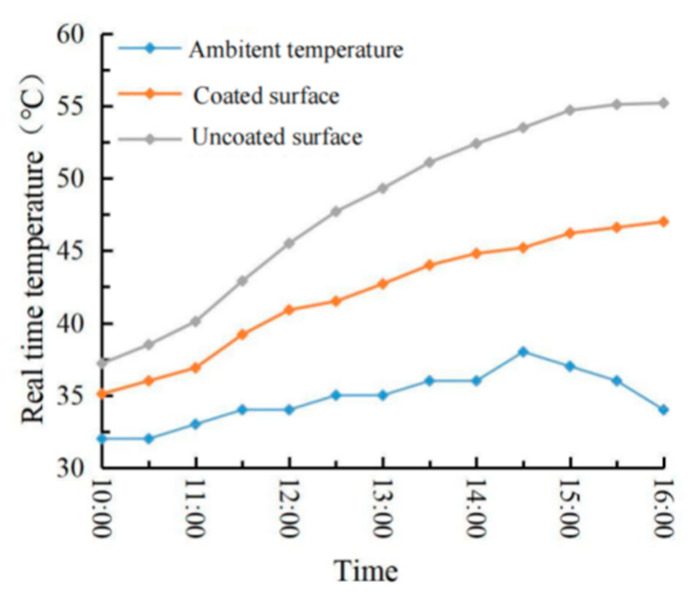
Real-time temperature chart.

**Figure 18:**
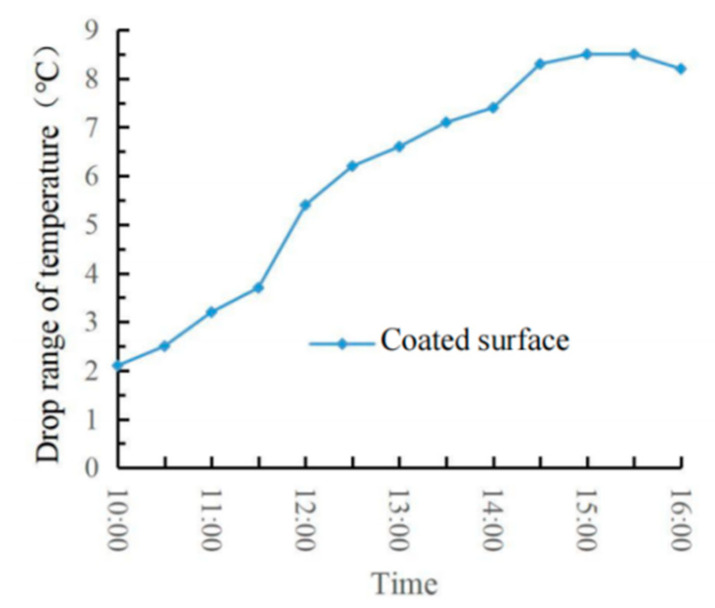
Pavement cooling value diagram.

The real-time temperature diagram and the road surface cooling value diagram are shown in Figure 17 and Figure 18. It can be seen from Figure 17 and Figure 18 that the temperature rise law of the two specimens was basically the same. Still, the temperature of the specimen with a coating was lower than that of the specimen without coating in each period. The cooling capacity of the thermal reflection coating gradually increased with the increase of the ambient temperature. The reason is that the solar radiation reflection and scattering speed that radiates to the coating surface are fast at low ambient temperature, so the temperature is relatively close. At 14:30, the ambient temperature reached the maximum of 38 °C, but at 15:00, the coating cooling value reached the maximum of 8.5 °C. At this time, the ambient temperature was 37 °C, the road surface temperature was 54.7 °C, and the coating surface temperature was 46.2 °C, with a cooling rate of 15.5%. Due to the limitation of time and place and other external factors, the ambient temperature did not reach above 40 °C, and the road surface temperature did not exceed 60 °C. Moreover, due to the interference of various external factors, such as wind speed and humidity, in the outdoor cooling test, the results were not consistent with those in the indoor cooling test. Therefore, the actual cooling effect of the thermal reflection asphalt pavement coating needs further certification.

**Figure 19:**
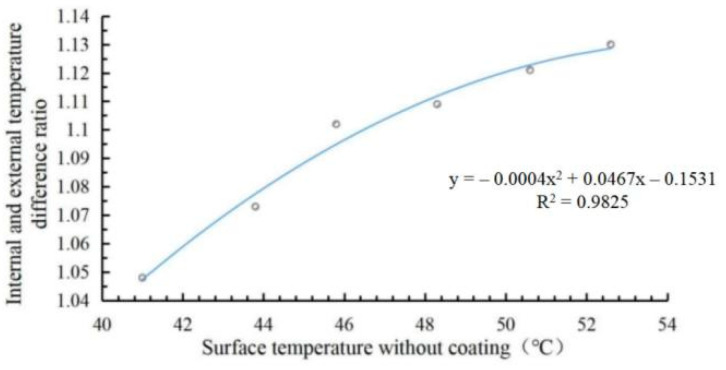
Trend chart of indoor and outdoor cooling value ratio.

Since the surface temperatures corresponding to each temperature measurement time point in the indoor and outdoor cooling tests are almost inconsistent, the cooling capacity value can be calculated according to the outdoor asphalt pavement surface temperature, and its cooling value under the that the surface temperature of the uncoated specimen in the indoor cooling test was consistent with that of the indoor cooling test. Then, the indoor cooling value is compared with the outdoor cooling value to obtain the internal and external cooling values, as shown in Table 8. With the surface temperature of the indoor uncoated rut plate as the horizontal axis and the cooling value ratio as the vertical axis, the trend line of internal and external cooling value ratio is shown in Figure 19.

Figure 19 shows the fitting curve of indoor and outdoor cooling values as follows:

(2) $$y=-0.0004x^{2}+0.0467x-0.1531(R^{2}=0.9825)$$

Formula: *y*—indoor and outdoor cooling value ratio; *x*—uncoated surface temperature, °C.

The maximum temperature of the uncoated surface in the indoor cooling test was 64.8 °C as the value, which was brought into Formula (2) to calculate the ratio of the indoor and outdoor cooling value ≈ 1.193 (retaining the three-digit decimal). Thus, the theoretical cooling value of the outdoor test at this time was T_2_ ≈ 13.4/1.193 = 11.23 °C, and the cooling effect was excellent. The correlation of this fitting curve *R*^2^ = 0.9825 indicates a good correlation, so it can be used to estimate the outdoor cooling value.

### 4.3. Anti-Slip Performance

The anti-sliding pendulum value and structural depth of the measuring points without coating and the measuring points with coating are shown in Table 9 and Table 10.

It can be seen from Table 9 that the anti-skid value of the pavement after the coating is reduced from 67 to 60 (a decrease of 10.4%). However, it still meets the requirements of the ‘Specification for Design of Highway Asphalt Pavement ’(JTG D50-2017) for the anti-skid value of the pavement (BPN ≥ 45). To reduce the skid resistance of asphalt pavement weakened by heat-reflective coating after laying, the coating selects ‘sandwich’ structure that is coating the bottom layer + anti-skid particles + coating surface coating structure. Soft anti-skid particles mainly include rubber and plastic particles of different textures. They have low hardness and certain elasticity. When used in the carriageway, they are often prone to deformation due to vehicle load, which makes them unable to provide greater friction. In addition, in the case of oil and water, the vehicle slip is not conducive to driving, but the effect is better when used on the sidewalk. Rigid anti-skid particles usually use inorganic material particles such as diamond, ceramic, quartz, and natural sand. This kind of anti-skid particles in contact with the rubber tire due to its high hardness led to a certain deformation of the rubber tire. Hence, the tire and the ground contact surface widened to better achieve the anti-skid properties. It was also easy to pierce the oil and water films to enhance the friction of the road in this state, and it is an excellent choice for anti-skid particles. However, ceramic particles are brittle, fragile, and not durable when used as anti-skid particles for heavy traffic. Quartz sand is low in cost and high in hardness. Therefore, quartz sand is selected as anti-skid particles in this paper, which also effectively ensures that the coating has good performance for a long time.

Table 10 shows that the structural depth of asphalt pavement after the coating is reduced from 0.80 mm to 0.66 mm (17.5%) but still meets the requirements of TD ≥ 0.55 mm in ‘Highway Engineering Quality Inspection and Evaluation Standard’ (JTG F80/1-2017). Therefore, although applying a thermal reflection coating on asphalt pavement weakened the anti-skid properties of pavement to some extent, it still greatly exceeds the minimum value in the specification, and the coating has a good anti-skid performance.

### 4.4. Wear Resistance Evaluation

The wear mass loss of each group of specimens is shown in Table 11. According to Table 11, the mass loss of the specimen coated with thermal reflection coating after wear meets the W < 0.2 g/m^2^ specified in the ‘epoxy resin surface coating material’ (JC/T 1015-2006), indicating that the thermal reflection asphalt pavement coating can reduce the wear degree of traffic tools on the road surface and has good wear resistance.

Because the indoor test environment was not necessarily consistent with the actual outdoor situation, the heat-reflective asphalt pavement coating was brushed on the asphalt pavement. The coating surface was observed after six months under the repeated load of the actual traffic participants and the erosion of the rain, snow, sand and stone, and the coating was brushed for two months, four months, and half a year. The structural depth and the anti-sliding values of the coating surface were tested and compared with those just brushed. The test results are shown in Table 12 and Table 13.

It can be seen that there are no obvious wear marks on the surface of the coating under the action of the external environment for six months. Table 12 and Table 13 show that with the increase in coating service time, the structural depth and anti-slipping values showed an increasing trend and then a decreasing trend. After half a year of painting, the structural depth of the coating decreased from 0.66 mm to 0.63 mm (with a decrease of 4.5%), and the anti-sliding value decreased from 60 to 57 (with a decrease of 5.0%). This is because when the coating is just brushed, the surface state of the coating changes from fuller and smoother to rougher when the wheel is rolled, which increases the structural depth and anti-skid value. Under the subsequent effects, the anti-skid particles will gradually be worn out or even fall off, resulting in a small decrease in both. However, in half a year, the overall change trend is small and meets the specification requirements, indicating that the coating also has good anti-sliding performance. After a wet-wheel wear test, the unit wear quality of the specimen coated with the coating solution meets the specification requirements. After six months of external action, the appearance of the coating had little change, and the anti-skid ability had little change, so the coating has good durability.

### 4.5. Evaluation of Impermeability

The seepage coefficients of five rut plate specimens before and after coating were compared, as shown in Table 14. It can be seen from Table 14 that the water permeability coefficient after coating was 0, while the average value of the water permeability coefficient before the coating is 147 mL/min, indicating that the coating has good water permeability resistance. The excellent water sealing effect after application to the pavement makes it difficult for water to penetrate into the structural layer in pavement damage. This is because part of the coating solution will gradually penetrate into the micropores and cracks of the pavement when it is not dried, and it plays a filling and sealing role after curing. Therefore, the thermal reflection coating plays an important role in preventing water damage, so it is also a type of pavement pre-curing material.

### 4.6. Anti-Aging Performance Evaluation

**Figure 20:**
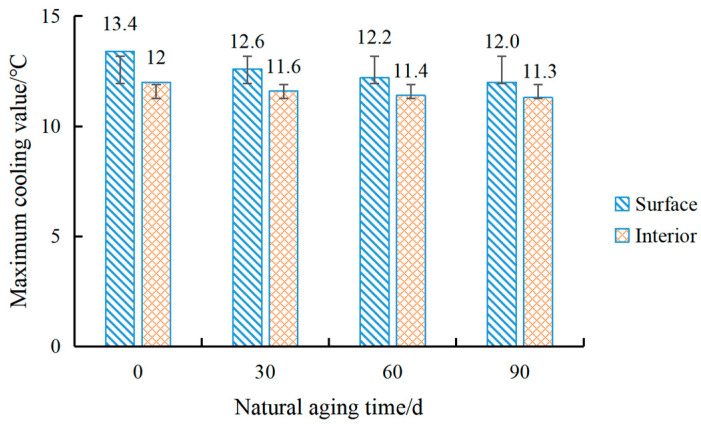
The cooling amplitude diagram of the coating after aging.

The heat-reflective coating will not be invaded and corroded by natural environmental factors all the time after service. Under the irradiation of ultraviolet light, most inorganic materials can maintain their stability, but organic materials will gradually age [23]. The epoxy resin used in this coating is an organic material, so the cooling capacity of the coating after outdoor aging was tested. The coating was painted on a rut board to test its cooling capacity and was placed in a natural environment after being hit by wind and rain, sun and rain. The cooling capacity was tested at 30 d, 60 d and 90 d, respectively. Its maximum cooling value is shown in Figure 20.

It can be seen from the above figure that the cooling effect of the coating gradually decreases with the continuous natural aging process. After 90 days, the maximum cooling values on the surface and inside are weakened by 1.4 °C and 0.7 °C, respectively. The main reason is that epoxy resin is a high molecular polymer, and its resistance to photooxidation is not too high. Ultraviolet light will break its chemical bonds, and the irradiation of infrared light and visible light will accelerate aging and degradation. Its chemical structure determines that under photothermal conditions, some chemical bonds in the molecule were highly active and easily generated free radicals for an oxidation reaction, which will affect the cooling performance of the coating [24]. However, the sericite powder and fumed silica added to the coating greatly delayed the oxidation and aging process of the coating, and the strong inertia of the titanium dioxide made the coating very stable. Therefore, after 90 days of natural aging, the cooling capacity of the coating did not suffer too much loss, and it still had a good cooling effect. At the same time, after March, the coating did not appear to have yellowed, become brittle and display any other aging phenomenon. The error line obtained by calculating the standard deviation shows that as time increased, the surface of the heat-reflective coating aged faster than the interior.

The gloss loss rate can also be used to measure the degree of aging of the heat-reflective coating. The irradiation of ultraviolet light will break the macromolecular chain to form a new molecular chain, which is the main reason for the change in the coating structure and, thus, the production of internal stress. When the internal stress accumulates, it can result in uneven cracks on the coating surface, which is intuitively reflected in the decrease of gloss. The gloss of the coating before and after aging was measured three times in parallel using a gloss meter, and the average value was taken. The gloss of the coating before aging was 32.6, and the gloss after aging was 30.8. The gloss loss rate was calculated by substituting it into Formula (3) [25]:

(3) $$\mathrm{Gloss}\;\mathrm{loss}\;\mathrm{rate}=\;\frac{A_{0}-A_{1}}{A_{0}}\times 100\%$$

In the formula:

A_0_-gloss measurement value before aging;

A_1_-Measured value of gloss after aging.

It can be seen that the light loss rate of the coating is 5.5%, which is a very slight light loss, indicating that the coating had a very small light loss rate after three months of natural aging and had strong aging resistance.

## 5. Conclusions

(1) A new type of asphalt pavement thermal reflective coating was developed. The emulsion mixed with epoxy resin and liquid phenolic resin was used as the matrix resin, and silica, hollow glass beads and sericite powder were used to replace part of titanium dioxide as the functional filler of the thermal reflection coating. Compared with all titanium dioxide as the functional filler, it was more economical, and the physical and chemical properties were more prominent.
(2) After surface modification of functional fillers, it was found that the particles were hydrophobic and lipophilic, which could be stably and uniformly dispersed in the thermal reflection coating of asphalt pavement and displays the cooling effect of the coating.
(3) With the cooling value, viscosity and gloss as the evaluation indexes, the orthogonal experimental design analysis was used to optimize the distribution ratio of each filler group that could best balance the three indexes as follows: 18 portions of modified TiO_2_, 3 portions of modified SiO_2_, 5 portions of modified hollow glass microspheres and 6 portions of modified sericite powder were added to each 100 mass portion of the mixed matrix resin, and the amount of pigment was determined to be 3% of the matrix mass after testing.
(4) The indoor cooling test showed that the optimal coating amount was 0.8 kg/m^2^, and the double-layer coating structure was adopted, i.e., the bottom layer was white pigment-free coating, and the upper layer was colored layer. The test results showed that when the coating amount of the bottom layer and the surface layer was 0.4 kg/m^2^, the coating structure could make the internal and external cooling values of the mixture reach 12.0 °C and 13.4 °C, respectively, and the cooling capacity increased by 11.1% and 8.1%, respectively, compared with the single coating structure. Due to the limitations of the external environmental conditions, the maximum cooling value of the outdoor cooling test is 8.5 °C, which is somewhat different from that of the indoor test. However, according to the fitting calculation of the indoor cooling test value, it can be seen that the outdoor asphalt pavement surface cooling value will be close to 11.23 °C when the ambient temperature is consistent, which has progressive achievements.
(5) The new asphalt pavement heat-reflective coating has good skid resistance, wear resistance, impermeability and aging resistance, indicating that the heat-reflective coating has good excellent prospects for application.

## Figures and Tables

**Table 1:** Material properties of non-solvent E51 (618) epoxy resin.

Resin Category	Epoxide Equivalent (g/EP)	Chromaticity (Platinum–Cobalt color)	Viscosity at 25 °C (mPa·S)	Hydrolyzable Chlorine (%)	Volatile Matter (%)
E51 epoxy resin	186.6	23	12,380	0.0623	0.087

**Table 2:** Material properties of 593 curing agent.

Category of Curing Agents	Appearance	Amine Value (mgKOH/g)	Density (g/cm^3^)	Active Hydrogen Equivalent	Viscosity at 25 °C (mPa·S)
593 epoxy curing agent	Colorless transparent liquid	500~600	0.985	45~48	150 ± 25

**Table 3:** Material properties of liquid phenolic resin.

Category of Curing Agents	Appearance	Amine Value (mgKOH/g)	Density (g/cm^3^)	Active Hydrogen Equivalent	Viscosity at 25 °C (mPa·S)
593 epoxy curing agent	Colorless transparent liquid	500~600	0.985	45~48	150 ± 25

**Table 4:** Main characteristics of four functional materials.

Name	Main Feature
Rutile titanium dioxide	It has strong light scattering ability, anti-ultraviolet ability, stability and refraction ability, high density, low photochemical activity, high refractive index and reflection ratio to near-infrared radiation, and can obviously shield solar thermal radiation energy.
Hollow glass microspheres	The coating has low density, low thermal conductivity, high reflection ratio, good thermal insulation, sound insulation, stability and flow smoothness. The addition of small doses of the coating has a low effect on the viscosity of the coating, so it can reduce the amount of solvent, reduce the emission of VOCs, and enhance the elasticity of the coating, which greatly reduces the possible cracks and shedding phenomena [20,21,22].
Silicon dioxide	It has porosity, heat insulation, high-temperature resistance, strong dispersion, high reflection and barrier ability to light and heat, and strong weather resistance. It can improve the viscosity and stability of the coating and also has a certain effect on the extinction of the coating.
Sericite powder	The lamellar structure can form the ‘maze effect‘ of heat transfer due to its large aspect ratio and significantly improve the UV shielding ability, impermeability, surface hardness and corrosion resistance of the coating. Moreover, due to the polarization effect of mineral crystals and the intervention effect of interlayer water molecules, the ability of the coating to shield ultraviolet, microwave and infrared rays is higher than that of all other inorganic fillers, so it can greatly improve the aging resistance and high-temperature resistance of the coating.

**Table 5:** Packing orthogonal design Table L9 (3^4^).

Level	Factor Dosage (Part)			
	TiO_2_	SiO_2_	Hollow Glass Microsphere	Sericite Powder
1	15	2	4	4
2	15	3	5	6
3	15	4	6	8
4	16.5	2	5	8
5	16.5	3	6	4
6	16.5	4	4	6
7	18	2	6	6
8	18	3	4	8
9	18	4	5	4

**Table 6:** Orthogonal test results of packing.

Test Number	Filler/ (Fill + Base)	Drop Range of Temperature (°C)	Kinematic Viscosity (mm^2^/s)	Glossiness
1	20.0%	12.7	159.9	34.1
2	22.5%	13.4	187.5	33.0
3	24.8%	13.6	226.4	33.6
4	23.9%	13.9	189.6	36.8
5	22.8%	13.9	208.8	28.7
6	23.4%	13.6	197.6	30.8
7	24.2%	14.2	205.3	33.6
8	24.8%	14.0	198.7	33.7
9	23.7%	13.9	203.8	26.7

**Table 7:** Components of each material of heat-reflective asphalt pavement coating.

Category	Name of the Material	Content (Mass Fraction)
Basal body	Solvent-free epoxy resin	97
	Liquid phenolic resin	3
	593 curing agent	25
Functional fillers	Modified rutile titanium dioxide	18
	Modified silica	3
	Modified hollow glass beads	5
	Modified sericite powder	6
Pigment	Iron oxide red powder	3
Assistant	692 epoxy active diluent	8
	Gas phase silica	0.5
	Propylene glycol	2.5
Anti-slip pellet	Quartz sand	5

**Table 8:** Indoor and outdoor cooling value ratio at the same temperature.

Surface Temperature of Indoor Rut Plate (°C)	The Cooling Value of Indoor Test T_1_ (°C)	The Calculated Outdoor Cooling Value T_2_ (°C)	Internal and External Cooling Value Ratio (T_1_/T_2_)
36.3	2.1	-	-
41.0	3.6	3.435	1.048
43.8	4.6	4.288	1.073
45.8	6.1	5.533	1.102
48.3	7.1	6.400	1.109
50.6	7.8	6.961	1.121
52.6	9.0	7.962	1.130
54.8	10.4	-	-
57.0	11.7	-	-
61.1	13.2	-	-
64.8	13.4	-	-

**Table 9:** Asphalt pavement anti-skid pendulum value.

Surface Condition	Pendulum Value (BPN)	Average Value (BPN)	Average Value Difference (BPN)
>No coating	70	67	7
	63		
	68		
Coated	61	60	
	60		
	58		

**Table 10:** Texture depth of asphalt pavement.

Surface Condition	Measuring Points	Tectonic Depth TD (mm)	Average Tectonic Depth (mm)	Average Difference (mm)
No coating	Point 1	0.79	0.80	0.14
	Point 2	0.82		
	Point 3	0.80		
Coated	Point 1	0.66	0.66	
	Point 2	0.64		
	Point 3	0.67		

**Table 11:** Quality loss comparison of wet wheel wear specimens.

Groups	Surface State	Wear Mass Loss (g·m^−2^)	Mass Loss Difference (g·m^−2^)
Group 1	Coated	0.166	0.073
	No coating	0.093	
Group 2	Coated	0.163	0.076
	No coating	0.087	
Group 3	Coated	0.162	0.071
	No coating	0.091	
Average value	Coated	0.164	0.074
	No coating	0.090	

**Table 12:** Comparison of texture depth.

Surface State	Tectonic Depth (mm)			Mean Value of Tectonic Depth (mm)	Mean Difference (mm)
When just painting	0.66	0.64	0.67	0.66	-
Two months later	0.67	0.68	0.67	0.67	+0.01
Four months later	0.65	0.63	0.64	0.64	−0.03
Half a year later	0.63	0.62	0.63	0.63	−0.01

**Table 13:** Comparison of anti-skid pendulum value.

Surface State	Anti-Skid Value (BPN)	Anti-Skid Average Value (BPN)	Average Difference (BPN)
When just painting	61	60	-
	60		
	58		
Two months later	62	61	+1
	60		
	62		
Four months later	58	58	−3
	59		
	58		
Half a year later	57	57	−1
	57		
	56		

**Table 14:** Results of water permeability coefficient.

Groups	Surface State	Permeability Coefficient C_W_ (mL/min)	C_W_ Difference (mL/min)	Average Difference (mL/min)
Group 1	Coated	138	138	147
	No coating	0		
Group 2	Coated	156	156	
	No coating	0		
Group 3	Coated	143	143	
	No coating	0		
Group 4	Coated	147	147	
	No coating	0		
Group 5	Coated	151	151	
	No coating	0		

## Data Availability

All data supporting this study’s findings are included within the article.
